# Impact of medical students' descriptive evaluations on long-term course development

**DOI:** 10.1186/1472-6920-6-24

**Published:** 2006-04-25

**Authors:** Mats Wahlqvist, Annika Skott, Cecilia Björkelund, Gösta Dahlgren, Kirsti Lonka, Bengt Mattsson

**Affiliations:** 1 Department of Primary Health Care, the Sahlgrenska Academy at Göteborg University, Sweden; 2 Department of Education, Göteborg University, Sweden; 3 Department of Applied Educational Sciences, University of Helsinki, Finland

## Abstract

**Background:**

In medical education, feedback from students is helpful in course evaluation. However, the impact of medical students' feedback on long-term course development is seldom reported. In this project we studied the correspondence between medical students' descriptive evaluations and key features of course development over five years.

**Methods:**

Qualitative content analysis was used. The context was consultation skills courses in the middle of the Göteborg undergraduate curriculum during five years. An analysis of 158 students' descriptive evaluations was brought together with an analysis of key features of course development; learning objectives, course records, protocols from teachers' evaluations and field notes. Credibility of data was tested by two colleagues and by presenting themes at seminars and conferences. Authors' experiences of evaluating the course over many years were also used.

**Results:**

A corresponding pattern was found in students' descriptive evaluations and key features of course development, indicating the impact of students' open-ended feed-back. Support to facilitators and a curriculum reform also contributed.

Students' descriptive feedback was both initiating and validating longitudinal course implementation. During five years, students' descriptive evaluations and teachers' course records were crucial sources in a learner-centred knowledge-building process of course development.

**Conclusion:**

Students' descriptive evaluations and course records can be seen as important instruments in developing both courses and students' learning. Continuity and endurance in the evaluation process must be emphasized for achieving relevant and useful results.

## Background

How can students' views be used for developing courses in the transition to clinical education? This question is discussed in recent reports of students' opinions and learning experiences in the transitional phase [1,2].

Since the days of Flexner, a conventional 20^th ^century version of the medical curriculum contain a preclinical phase of biomedical sciences followed by a later clinical phase [3]. To students, the transition from the first phase to the second forms a critical stage in which the term 'shock of practice' has been introduced to characterize students' learning problems [4]. Even though many curricula have undergone changes in order to integrate preclinical and clinical studies, the shift and the overall template still have a large influence. Students perceive the transition as a stressful part of the curriculum [5]. On basis of these results, an extensive introduction and increased support for students are suggested to be explored in the future [1,2,5].

On a course level, students' course descriptions or portfolios might be a suitable instrument for monitoring changes of the transition. Students' evaluations are often used for developmental purposes. However, administrative purposes are also involved and these aims need to be separated from the developmental purposes [6,7]. Scientific reports on the importance of students' evaluations are however scarce and in a review of student evaluations, researchers in higher education states "there is very little research evidence on the usefulness of students evaluations in improving any of the processes thought to benefit from them" [8]. Moreover, processes in course development are often complex and are difficult to analyse. Attempts to study the role of students' evaluation feedback in long-term course development and implementation are seldom reported in medical education, there is only one match found in Pub Med [9].

In this study we intend to analyse the impact of students' descriptive feedback in the transition to clinical education during five years of factual course development.

### Context

In 1993, a nine-week clinical introduction course 'Consultation Skills' started in the Göteborg undergraduate curriculum. The consultation was launched as a unique concept by British researchers in the 1980' [10]. A patient-centred approach in communication is an essential part of the concept [11]. In the course, students' first encounter between patients and physicians took place in general practice and was integrated with learning clinical examination skills, medical psychology and medical ethics [12]. Learning in small groups and learning in practice together with facilitators and clinical supervisors was implemented. Students met facilitators in four settings: in primary health care centres; in an art-of-interviewing week using the Kagan video method [13]; in geriatric wards and in continuous small groups of skills training and reflection. In cooperation with the three leaders of the course, the first author (MW) was working as a course assistant, coordinating group facilitators, organizing and evaluating courses. AS and CB were two of three course leaders. At the beginning of course implementation, extensive formats were used consisting of structured evaluation items covering the particular parts of the course.

In 1995, two years later, we felt more familiar with the new context and searched for a student-centred, global and uncomplicated evaluation approach. Two of the authors (CB, MW) attended university courses of learning in higher education. Qualitative research methods and a local study on course evaluations inspired in trying unconventional descriptive evaluation methods [14,15].

The general idea was to adapt and harmonize the evaluation idiom to the central knowledge of the course. Student-centred descriptive evaluations would better correspond to the patient-centred approach learned in the course. Instead of asking for structured, closed and short answers, we invited students to expand on their learning experiences during the course. A very substantial student evaluation in the form of a letter also contributed to the shift. This meant that we deliberately used evaluations primarily as a *tool for listening and understanding *in course development. Consequently, the purpose of students' evaluations shifted, from mapping values of particular events to grasping students' learning experiences in the course as a whole.

**Figure 1:**
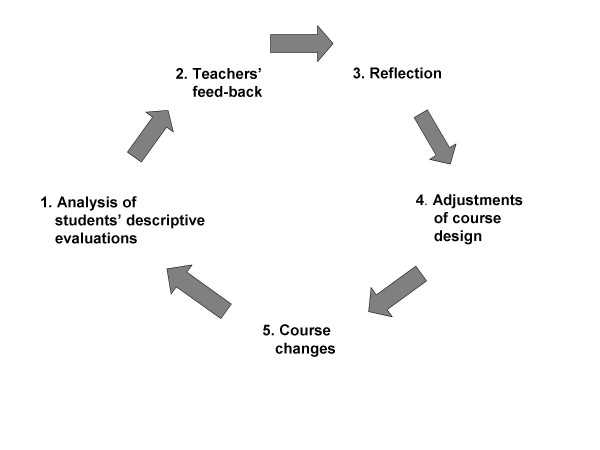
**Evaluation cycle in course development**. Evaluation cycle of teachers' systematic course evaluation and development. Consultation skills course, Medical faculty, Göteborg university.

After each course, the documentation including teachers' views and reflections were also collected, forming an evaluation cycle in course development (Fig 1). Thus, two sources of rich data were available; students open-ended descriptions and course documentation.

The aim of the project was to study the correspondence between students' descriptive evaluations and key features of course development over five years.

## Methods

The study started in 1995 and covers five years of course development.

### Participants and materials

Five hundred and thirty seven medical students attended nine Consultation Skills courses during the study period.

#### Materials from students

During the last day of the Consultation Skills course, students were asked to anonymously answer only one open-ended question:"What do you think of the course in Consultation Skills, with reference to the course design and examination? "From spring 1996, a tick box for marking students' gender was included in evaluations.

#### Materials from teachers and course organizers

Materials from teachers were course records and documents; plan of learning objectives, course schedules, teachers' schedules and assessment guides; teachers' protocols from systematic evaluations after each course and weekly protocols from course team meetings.

### Procedures

Students were given 45 minutes of the last part of the course schedule to finish their evaluations. The evaluation question was attached to two blank sheets of paper and was distributed to students in a lecture room. Most students spent over 30 minutes writing their evaluation.

### Data collection

#### Data from students

Four evenly distributed courses over the five-year period produced 158 evaluation stories that were used as sample. Student data consisted of 214 pages of written text from autumn 1995, spring 1996, spring 1997 and autumn 1998. (Table 1). Gender proportions of the whole material were retained.

#### Data from teachers

Records and documents described under Materials from a series of courses were included as data: autumn 1995, spring 1996, spring 1997, autumn 1998 and autumn 1999. In all, course documents comprised about 250 pages of text.

Six weeks after termination of a course, results from students' evaluations were presented in follow-up seminars. The work of facilitators and teachers was also discussed in small groups and followed by reflection. Evaluation results were collected and discussed by the course committee and after critical reflection; some were implemented in the next course. The course committee met once a week throughout the years and notes were taken.

### Data analysis

#### Step one. Analysis of students' descriptive evaluations

A content analysis of text was performed [16,17]. Data were analysed twice; first as part of teachers' immediate evaluation analysis after each course and then in a retrospective more thorough analysis. In the immediate analysis, evaluations were coded by writing core quotations, addressing main teaching and learning events and students' experiences. Similar citations were summarized and major patterns of students' statements and course experiences were identified. Recurrent themes emerged from these preliminary analyses.

The research analysis of students' descriptive evaluations was performed in 2002–2005 and consisted of the following steps:

1. Answers were first thoroughly read in extenso, reaching for a global understanding of the content in each student's course story.

2. Students' statements were coded into general categories.

3. Tabulations were also used in order to encompass an overall picture of students' statements in their course descriptions.

4. Categories were established on basis of 2 and 3.

5. Main themes were condensed.

6. Deviant cases of students' statements not fitting main themes were identified.

#### Step two. Analysis of key features of course development

Key features in course development were identified by content analysis of a series of course records and documents (see above). The text material was abundant. Therefore, Biggs' framework of main components in university education (learning objectives, teaching and learning activities, assessment, learning climate and institutional climate, rules and procedures) was used [18]. Thus, changes of main course components were focused in the analysis and a template style was used [19,20]. It implied that data were arranged according to the framework, units were identified, and the achieved material was read several times in close chronologic comparison. Finally, key feature themes were formed.

#### Step three. Correspondence between main themes of students' descriptive evaluations and key features of course development

In the last, third step, results from content analysis of students' descriptive evaluations and content analysis of key features of course development were brought together. By these measures a corresponding pattern was searched for, thus reflecting the impact of students' open-ended evaluations on course development.

To increase reliability and concordance of the three steps of analysis, AS and CB who participated in course development read half of the evaluations, assessed and checked analysis themes. Interpretations were discussed by main author and co-authors AS and CB during several meetings. Initial data was expanded and after re-analyses, concordance was reached. Theoretical perspectives used in interpretation were an educational learner-centred perspective and patient-centeredness in medicine. The trustworthiness of data was further tested at a seminar by two external assessors from the medical faculty and at a Nordic conference of education and research in medical communication. The assessors and research colleagues recognized our main experiences and it seems as if our findings are transferable to their context.

## Results

Students' descriptive evaluations and records of course documentation presented a very rich and varied material. Results of the three steps of analysis are presented below. The third step analyse the correspondence between students' descriptive evaluations and key features of course development over five years.

### I Analysis of students' descriptive evaluations

Seven main themes emerged in the analysis of students' descriptive evaluations. A framework of the content analysis is presented in Table 2. Main themes and categories supporting their establishment are presented below.

#### At last, learning professional skills in practice

##### Learning goals articulated

Some students articulated basic learning goals; how to interview patients, how to perform a physical examination and how to write a medical record.

##### Active learning in practice

Most students were eager to express the importance of meeting GPs and to be trained in physical examination skills. Consultation training was specially appreciated in primary care.

##### Missing professional training in earlier medical studies

A rather harsh tone of disappointment was present in some of these statements of students' learning experiences in earlier medical studies. Expressions used were e.g. "cramming" and an "anonymous" or even "inhumane" learning environment.

#### To be active and to have a choice

##### Course content does not fit to lectures

Some students expressed that the content in lectures of consultation skills and issues of the professional role would be more suitable for interactive group discussions.

##### Compulsory didactic activities criticized

In the first parts of the material compulsory activities were put in question and a clear protest could be traced. This category vanished after changes in 1997 to a reduction in compulsory activities.

#### Lack of unity

##### Miscellaneous course content

Some students were confused by too many different learning activities, not finding "the red thread" of the course.

##### Uniform guidelines to facilitators wanted

These statements were complaints of different standards in training of physical examination skills and assessment.

#### Course design works

##### Mix of practice and theory

Students made a positive remark on the course design's mixture of practice training and didactic modules. Some were surprised that their fast introduction to patient encounters in primary care turned out so well.

##### New ways of learning

New ways of learning in new settings compared to earlier studies were mentioned. In some of these statements, students were astonished that they became so emotionally tired from clinical experiences.

#### Authentic and relevant practical examination

##### Practice examination a learning experience

The practical examination was appreciated throughout the study period. Some students expressed satisfaction of their growing clinical competence shown in the examination.

##### Acceptance of examining reflections on practice experiences

From 1997, students were asked in a home essay assignment to reflect on observed consultations in primary care and to use conceptions of core learning objectives. Students admitted that they were initially hesitant but were later surprised that the home essay served its purpose.

#### Support and encouragement from facilitators

##### Support

Students expressed that they were well received by facilitators in practice and appreciated that facilitators were student-centred.

##### Encouragement

Students were challenged by facilitators to perform new tasks for which they were highly motivated. A sense of optimism and hope is clearly present in these statements.

#### Awareness and confidence

##### More aware of patient's perspective

These statements were conveyed as a consequence of events and experiences during the course.

##### Involvement and interaction

A combination of personal involvement and team work were elicited in statements of an art-of- interviewing week and of small group work.

##### Self-reflection and confidence

These reflecting statements express that the awareness and the confidence in becoming a physician had changed, often in comparison to experiences in earlier stages of medical education.

#### Deviant cases

The deviant cases of the analysis displayed predominantly minimal and general remarks. An example is the statement 'Very good' as an evaluation of the whole course. Some cases not fitting into main themes were seen in spring 1996 and consisted of extensive comments on a debate held in a single lecture.

### II Analysis of key features of course development

Analysis of key features of course development is depicted in Table 3.

As seen from Table 3, according to Biggs' framework, a process of development occurred during the study period with respect to major course components. Active learning in practice was enhanced and student's degree of choice was strengthened. Consultation and clinical skills were identified as core learning objectives in a process of concentration. Education of facilitators in core learning objectives, including an introduction of a practice assessment guide, enabled assessment in context of core learning objectives. Of great importance in course development was the structured support and education of facilitators in 1997. Together with increased continuity, student-facilitator relationships and reflection were reinforced. External influences are seen in the last theme, a curriculum reform in 1996 implied reorganization and concentration of the course. Consequences of the curriculum reform were larger volumes of students and comparatively less teachers, doubled student courses during one semester and coordination with a course in clinical pharmacology.

There were also dead ends and drawbacks in course development. Many of them were due to over-ambitious goals and efforts to cover too much content. Thus, learning from less successful events was also an important part of course development and applies in particular to the key features 'concentration' and 'core learning objectives'.

### III Correspondence between main themes of students' descriptive evaluations and key features of course development

Main themes of students' descriptive evaluations and key features of course development are brought together and depicted in Table 4.

A corresponding pattern is seen in main themes of descriptive evaluations and key features of course development. A number of observations support this relation.

Students' reported urge to learn professional knowledge and skills mirrors that these areas being gradually selected and communicated as core learning objectives. In addition, a lack of unity of course content perceived by the students corresponds with education of facilitators in core learning objectives. Students' main theme 'To be active and to have a choice' corresponds to themes 'Active learning in practice enhanced' and 'Student's degree of choice strengthened' in course development. Assessment in context of core learning objectives matches students' perception of an authentic and relevant examination. The pattern of correspondence indicates that students' descriptive evaluations had an impact on course development.

Since course development occurred in a process over five years, it is possible to discern reciprocity. Students' evaluations affected course development and course development also affected students' evaluations. Thus, structured support and education of facilitators parallels students' main theme 'Support and encouragement from facilitators'. Similarly, reinforcement of student-facilitator relationships might be associated with students' theme 'Awareness and confidence'.

In addition, external factors contributed to course development. A curriculum reform in 1996 implied reorganization and concentration of course content. Another external influence was input of educational knowledge; from local scientific evaluations of the Göteborg curriculum and attending university courses of learning in higher education [14,15].

## Discussion

In an evaluation of a course in the transition to clinical education, students' descriptive evaluations and teachers' course documentation was analysed. A corresponding pattern of students' descriptive evaluations and key features of course development was seen in several dimensions, indicating the impact of students' feed-back on course development.

### Comments on method

In three consecutive steps of analysis, a quite voluminous text material covering five years of systematic evaluation and course development was brought together and condensed.

Limitations of the qualitative method in our study concerns credibility of data and the analysis method. Credibility is increased by conveying the analysis performed and the theoretical perspective used in interpretation [16,20]. Credibility was also achieved by many short and recurrent evaluation cycles during five years (Fig 1). Several learner-centred evaluation loops were thus forming a spiral of increased knowledge. The process has several similarities to action research, recommended as a potentially productive method for improving practice in medical education [21]. In order to ensure reliability, fifty percent of course documentation and students' evaluations respectively were read by two authors. In addition, main authors' participation and involvement in course development might have disturbed the trustworthiness of information obtained. Four strategies were used to balance this risk. First, in order to increase awareness and reflexivity, main author's preconceptions were clarified in writing before analysis [20]. Second, the theoretical perspective used in interpretation was presented and third; two co-authors read and checked analyses of data, resulting in expansion of data and re-analyses. Finally, credibility was tested by external assessment in seminars and in a Nordic research conference.

Research on student ratings of university teaching has been thoroughly reviewed by Marsh [22]. University student ratings are reported as quite reliable and reasonably valid and that useful information can be obtained in ratings. In educational research, as well as in medical practice, the utility and functional aspect is highly relevant [23]. Even though our research method is different from structured ratings, the functional aspect and the relevance to colleagues in medical education is similar.

Another concern may be the questions put in evaluations. Some students might have been restricted by asking about their thoughts of the course, course design and examination. The opening question could perhaps have been even more open-ended to cover a wider spectrum of student's learning experiences. However, the material we received in descriptive evaluations was rich and many facets of students' personal experiences and thoughts emerged spontaneously in statements, thus transforming students' evaluation feed-back into a "legible choir of students' voices". Despite the restricting components, we think the question was open enough to yield useful feed-back in course development.

Content analysis as a method of analysing evaluation data is discussed by Lincoln & Guba [24]. They separate analysis of manifest content and interpretation of latent content and argue the validity of interpreting content which is latent in documents and records. In our analysis of students' evaluations, the last phase of forming themes used latent content to some extent but the emphasis was put on analysis of manifest data. A combination of a scientific approach with context knowledge from clinical practice was required in the interpreting process. Listening to students was prioritized and that could be applied to doctors listening to patients.

Group dynamics may represent another source of bias. Student-teachers relationships in medical education are not symmetrical [25]. Enlarged group reactions to e.g. the compulsory requirements for passing the course should be appreciated as a potential source of distortion in data.

#### Comments on selection

The student sample was collected from four evenly distributed courses in order to avoid accidental results. Students in the study were not different from other courses in terms of student characteristics and passed/approved rates. In a Scandinavian context of undergraduate medical education, a mean response rate of 70% of voluntary evaluations, obtained over four years, seems acceptable [26]. However, the relatively low (46%) response rate in 1997 should be noted. The reason for the low 1997 response rate remains unclear, but a course party held the night before might be a factor to consider. On the other hand, gender proportions are retained and the overall response pattern in 1998 students (70% response rate) was similar to 1997 students. Our assumption is that students who actively chose to respond were more positive to the course than non-responders.

### Comments on results

Students descriptive feed-back was both initiating and validating a longitudinal process of course development and implementation. Through the analysis, an overview of five years of this process was achieved. A corresponding pattern between students' descriptive evaluations and key features of course development was found.

An eagerness for professional training was evident in students' descriptive evaluations. The analyses enabled a deeper understanding of students' perceptions of learning conditions and resulted in a selection of core learning goals. It emerged that hands-on training of clinical examination skills is pivotal in the transition to clinical education, forming a rite of passage in student's professional socialization process. A student's self-image as a future physician appears to depend on learning doctor's professional skills [5]. The concept of the consultation acknowledges the clinical context and learning examination skills seems to enhance motivation for learning consultation skills in the transitional period [12].

An important part of the result are the corresponding themes 'To be active and to have a choice' and adherent changes carried out in course development. From students' point of view, the correspondence of these themes may be interpreted as signs of an empowering process in which students' voices were heard. The examination in an authentic setting was clearly appreciated throughout the study period and corresponded in course development to assessment of core learning objectives and extended time for feed-back. In fact, a change of the examination was the main tool to achieve clarity of learning goals in relation to students. An obvious linkage of learning objectives, learning activities and assessment was organised and communicated [18]. Introduction of a transparent assessment guide to practice examination seems to have translated learning objectives into comprehensible performance tasks for students. By this move, group facilitators also became more aware of core learning goals since they were involved in assessment.

Interestingly, students theme 'Awareness and confidence' might be associated with the key feature 'Student-facilitator relationships and reflection reinforced' (see Table 3 and 4).

A student-teacher learning relationship including reflection has been asked for in the transitional period and in professionalism curricula [1,2,5,27,28]. Our course development and experiences support these assertions.

#### The role of external influences

As mentioned earlier, external factors affected the results. Biases seen here are organizational factors and bias from input of 'external' learning. A curriculum reform was implemented in the middle of the study period and due to shortage of personnel, a reorganization and concentration was necessary. However, these changes were well informed and guided by knowledge acquired from systematic analyses in course development. Furthermore, cooperation between the Curriculum Committee of the Medical Faculty and researchers in the Department of Education at Göteborg University played an important role. One of the authors (AS) chaired the Curriculum Committee of the Medical Faculty and another author (CB) was a member of the committee during the study period. These circumstances probably contributed to a climate positive to educational development and to implementation of the Action Programme in Education of Göteborg University [29]. Moreover, new perspectives from teachers' courses of learning in higher education and a scientific educational report on students' evaluations inspired us to try a new, unconventional approach of course evaluation [14,15]. Thus, aside of 'internal' learning from students' descriptions, input from 'external' knowledge of learning in higher education gradually increased course leaders' educational competence. An active intention and financial support to the initiation of the Consultation Skills course by the Curriculum Committee of 1989–1995 were also important factors in the overall course development.

## Conclusion

Students' descriptive evaluations and teachers' course records were helpful in a long-term evaluation forming a learner-centred knowledge-building process. Students' feedback was both initiating and validating longitudinal course implementation. Asking students to write an individual course description appears to bring a sense of mutuality to evaluations and create a new understanding of how students' learning events were experienced in the whole of a course. Evaluators and course organizers should also consider organizational factors affecting course development. Continuity and endurance in the evaluation process must be emphasized for achieving relevant and useful results. In conclusion, students' descriptive evaluations and can be seen as important instruments in developing both courses and students' learning.

## Competing interests

The author(s) declare that they have no competing interests.

## Authors' contributions

MW acted as the principal researcher, collected data and performed analyses. BM substantially contributed to conception and design of the study and revised the manuscript. AS and CB made substantial contributions in interpretation of analyses performed, in revising the manuscript and in the acquisition of data. GD and KL contributed to conception and design of the study and in revision of the manuscript.

## Pre-publication history

The pre-publication history for this paper can be accessed here:



## Figures and Tables

**Table 1:** Study sample. Students and course evaluations, *n *= 158. Autumn 1995, spring 1996, spring 1997, autumn 1998. Consultation skills course, Medical faculty, Göteborg university.

	**1995**	**1996**	**1997**	**1998**	**Total**
**Students (n)**	58	60	67	52	237
**Female (%)**	27 (46)	33 (55)	36 (53)	28 (53)	124 (52)
**Male (%)**	31 (54)	27 (45)	31 (47)	24 (47)	113 (48)
**Descriptive evaluations (n)**	41	46	31	40	158
**Response rate**	73%	74%	46%	77%	70%
**Female responders (%)**	n a	27 (59)	19 (61)	20 (50)	66 (52)
**Male responders (%)**	n a	19 (41)	12 (39)	20 (50)	44 (48)

N a = not available in 1995

**Table 2:** Main themes of students' descriptive evaluations. Framework of content analysis of students' descriptive evaluations (n = 158), Consultation skills course 1995–1998, Medical faculty, Göteborg university.

**Elements of meaning (examples)**	**Categories**	**Theme**
"I have learnt to talk to patients and physical examination skills...and I will develop later..." "...a "kick" at last to meet patients, happy for the first time since I started my studies... now I want to learn for life and not just the exam..." "...at last! after two years inhumane preclinical studies, a course that shows what being a doctor is about..."	learning goals articulated active learning in practice missing professional goals in earlier medical studies	**At last, learning professional skills in practice**

".... many lectures seemed to be interesting but they did not turn out well...more group discussions instead" "... what's the point in forcing us to attend to compulsory didactic activities?"	course content does not fit to lectures compulsory didactic activities criticized	**To be active and to a have a choice**

".. well, I found the course design, with both practice and theory, fairly good..." "...no rote learning now, but long days, I felt tired in a new way..."	mix of practice and theory new ways of learning	**Course design works**

"...too many small different things brought together..." "...my facilitator emphasized some items in the physical exam but the assessor did not think they were that important..."	miscellaneous course content uniform guidelines to facilitators wanted	**Lack of unity**

"... to have a practical examination is necessary and instructive...", "...the examination with patient was excellent, no stress and well organized..." "...examination: I feel secure in history and physical examination...", "...excellent that you take care of the follow-up if you should fail..." "...reflective home essay on experiences in practice: good questions...they were quite voluminous but gave me a possibility to deepen in a wider area...", "...I was surprised: the home essay was all right..."	practice examination a learning experience accept of examining reflections on practice	**authentic and relevant examination in practice**

"...group facilitators made a very good job, great support..." "...facilitators (in primary care) gave me a lot of support and help. We were encouraged to take own initiatives and learnt enormously during these days!"	support encouragement	**Support and encouragement from facilitators**

"...the course gave insight into how I am and think and especially how the patient thinks..." "Learnt enormously, including about myself...", "...we were asked to express our opinions and feelings..." "...first now I realize how much I have learned..." "...so many personal reflections, I feel more confident in my future role in health care...", "I feel strengthened and look forward to continue my medical studies..."	more aware of patient's perspective involvement and interaction self-reflection on learning, confidence	**Awareness and confidence**

**Table 3:** Key features of course development 1995–1999. Content analysis using Biggs' structure of major course components in university education. Consultation Skills course, Medical faculty, Göteborg university.

**Major course components (Biggs)**	**1995**	**1996**	**1997**	**1998**	**1999**	**Key features of course development**
**Learning objectives**	extensive		core: consultation and clinical examination skills communicated and clearly connected to assessment			*Consultation and clinical examination skills communicated as core learning objectives* *Facilitators educated in core learning objectives*

**Teaching and learning activities**	lectures, theme modules small group learning skills learning in practice		lectures reduced, compulsory themes made optional enhanced small group learning learning in practice emphasized			*Student's degree of choice strengthened* *Active learning in context enhanced*

**Assessment**	bi-modal		multi-modal reflective home assignment introduced and developed practice assessment guide made transparent extended time for feedback in practice examination			*Assessment in context of core learning objectives*

**Learning climate, relationships**	short learning relationships supervision of facilitators		continuity, longer learning relationships structure and support to facilitators: supervision, schedules, colloquiums, input of educational knowledge			*Student-facilitator relationships and reflection reinforced* *Structured support and education of facilitators*

**Institutional climate, rules, procedures**	support from curriculum committee, education		curriculum reform: increased workload, shortage of personnel			*External influence: curriculum reform implied reorganization and concentration*

**Table 4:** Correspondence between main themes of students' descriptive evaluations and key features of course development. Consultation skills course, 1995–1999, Medical faculty, Göteborg university.

**Main themes of students' descriptive evaluations**	**Key features of course development**
At last, learning professional skills in practice	Consultation and clinical examination skills selected and communicated as central learning objectives
Lack of unity	Facilitators educated in core learning objectives
To be active and to have a choice	Active learning in practice enhanced
	Student's degree of choice strengthened
Course design works	-
Authentic and relevant examination	Assessment in context of core learning objectives
Support and encouragement from facilitators	Structured support and education of facilitators
Awareness and confidence	Student-facilitator relationships and reflection reinforced
-	External influence: Curriculum reform implied reorganization and concentration
